# Pharmacological effect of *Argyrolobium roseum* (Camb.) Jaub & Spach extracts against lead‐induced toxicity in rats

**DOI:** 10.1002/fsn3.3570

**Published:** 2023-07-24

**Authors:** Naeem Rasool, Muhammad Ovais Omer, Aqeel Javeed, Muhammad Nawaz, Muhammad Imran, Muzzamal Hussain, Zarina Mushtaq, Entessar AL Jbawi

**Affiliations:** ^1^ Department of Pharmacology and Toxicology University of Veterinary and Animal Sciences Lahore Pakistan; ^2^ Department of Microbiology University of Veterinary and Animal Sciences Lahore Pakistan; ^3^ Department of Food Science and Technology University of Narowal‐Pakistan Narowal Pakistan; ^4^ Department of Food Sciences Government College University Faisalabad Faisalabad Pakistan; ^5^ Agricultural Extension Directorate, MAAR Damascus Syria

**Keywords:** anti‐inflammatory activity, antioxidant enzymes, aqueous and ethanolic extract, *Argyrolobium roseum* (Camb.) Jaub & Spach, Pb‐induced toxicity

## Abstract

*Argyrolobium roseum* (Camb.) Jaub & Spach (*Papilionaceae*) is a medicinal plant, cultivated in northern areas of Pakistan. The consumption of trace minerals (lead) is very toxic to the vital organs of the body, therefore the overcome of these minerals is very necessary. In this regard, this study aimed to assess the potential pharmacological effect of aqueous and ethanolic extract of *Argyrolobium roseum* (Camb.) Jaub & Spach against pb‐induced oxidative stress, histological changes in Pb‐induced rats' liver and kidney, and anti‐inflammatory effect. The metal concentrations in liver and kidney homogenates were measured through atomic absorption spectrophotometer. The antioxidant activity was measured through DPPH and FRAP assay. Pb concentrations were significantly higher in liver and kidney homogenates after injection of Pb acetate was given intraperitoneally (45.2 ± 6.8 and 58.8 ± 7.9, respectively; *p* < .0001). The level of Pb in liver and kidney homogenates was significantly reduced by aqueous and ethanolic extracts of *Argyrolobium roseum* (Camb.) Jaub & Spach. The Pb + Aq‐600 mg/kg‐treated rats exhibited a protective effect on hepatocytes cells against Pb‐induced liver injury and restored the cells of the kidney. Pb + Aq‐600 mg/kg showed higher antioxidant activity as compared to other treated groups. The highest decreased MDA level was found in liver and kidney homogenate of Pb + Aq‐600 mg/kg rats (11.2 ± 1.51 nmol/mg; *p* < .001) and GSH and CAT levels tended to normal after treatment of Pb + Aq‐600 mg/kg in rats. The ALAD, ALT, AST, and ALP level were enhanced and tended to be normal after the Aq‐400 and Aq‐600 mg/kg treatment in Pb‐exposed rats. The result showed that 600 mg/kg Aq + Pb exhibited significant (*p* < .001) anti‐inflammatory activity. The findings of this study concluded that treatment of the aqueous extract of *Argyrolobium roseum* (Camb.) Jaub & Spach reduces the renal and hepatic damage in Pb‐induced rats and it also decreases oxidative stress via improving antioxidant components.

## Graphical abstract

The current study aimed to evaluate the therapeutic effect of aqueous and ethanolic extracts of *Argyrolobium roseum* (Camb.) Jaub & Spach against Pb‐induced toxicity in rats. The findings of this study show that Pb inductions damage the kidney and liver tissues, and caused severe oxidative damage and functional disturbance due to accumulation in these organs of rats.

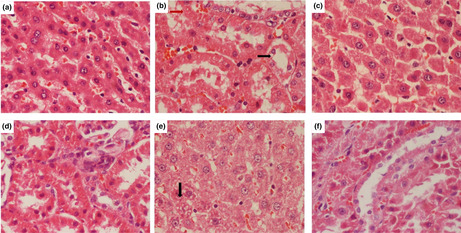

## 1 INTRODUCTION

Lead (Pb) is the most widespread and one of the major insidious environmental toxins like metal that is detected in all environmental and biological systems (Machida et al., 2009). This metal investigation indicated that Pb exposure on mammals reported to be devastating effects as it may become part of all abiotic factors of the environment such as dust, soil, brass, plumbing fixture, water, housewares, and lead‐mixed imported products. Therefore, its use in industries as well as for domestic purposes causes a huge threat to normal human health and laboratory animals (Kiran Kumar et al., 2009).

Now the small quantity of Pb exposure is harmful to humans and other mammals (Xia et al., 2010), including the generation of free radical‐mediated reactive oxygen species (ROS), which directly imbalances the pro‐oxidants and the antioxidants in the body that leads to oxidative stress (Franco et al., 2009). Its toxicity causes serious damage to important macromolecules, particularly to proteins, lipids, and nucleic acid. It also causes hepatotoxicity, neurotoxicity, nephrotoxicity, and genotoxicity in people, animals, as well as plants (Xia et al., 2010; Xu et al., 2006). Therefore, some scientists approach Pb toxicity through the use of ethnomedicines. *Artemisia absinthium* extract has been used to restore the antioxidant enzymes in the brain of Pb‐induced rats (Kharoubi et al., 2009). Ethanol extract *Aquilegia vulgaris* was used by El‐Nekeety et al. (2009) to improve the histological characteristics of the liver and kidney in Pb‐induced mice.

*Argyrolobium roseum* (Camb.) Jaub & Spach is a rare herb belonging to the family Papilionaceae. It grows in the tropical and temperate regions of Pakistan, India, Bangladesh, Nepal, Madagascar, South Africa, and Arabia (Sharma et al., 2016). It has a weak stem, ascending branches, and trifoliate leaves (Ram et al., 2007). *Argyrolobium roseum* (Camb.) Jaub & Spach has historically been used to treat a variety of skin conditions, including boils and scabies, as well as hepatitis, fatigue, and stomach and bladder infections (Abbasi et al., 2009). Ahmad et al. (2013) identify several flavonoids and phenolics from *Argyrolobium roseum* (Camb.) Jaub & Spach. The current study aimed to evaluate the therapeutic effect of aqueous and ethanolic extracts of *Argyrolobium roseum* (Camb.) Jaub & Spach against Pb‐induced toxicity in rats, and to evaluate the potential effect of extracts of *Argyrolobium roseum* (Camb.) Jaub & Spach against pb‐induced oxidative stress, histological changes in Pb‐induced rats, as well as liver and kidney and anti‐inflammatory effect.

## 2 MATERIALS AND METHODS

### 2.1 Experimental site

The present study was conducted in the Department of Pharmacology and Toxicology, and quality operational laboratory, UVAS, Lahore. All the experimental designs and procedures were approved by the Ethical Review Committee of UVAS, Lahore.

### 2.2 Plant material and extraction preparation

*Argyrolobium roseum* (Camb.) Jaub & Spach was obtained from the local market of Lahore city, Pakistan. The specimen of *Argyrolobium roseum* (Camb.) Jaub & Spach was identified by the Department of Botany, GCUF, Pakistan. The voucher sample was prepared and deposited in the Department of Botany, GCUF, Faisalabad, Pakistan. After washing with sterile distilled water, *Argyrolobium roseum* (Camb.) Jaub & Spach was placed under running tap water. The plant was then air‐dried and ground into a fine powder using a clean household grinder. The acquired powder was then put to use in further analysis. The extraction, screening, and characterization were executed following the guidelines described by Chauhan et al. (2011).

### 2.3 Evaluation of total phenolics and flavonoids content

The total phenolics and flavonoid contents were determined by methods as described previously (Piccolella et al., 2008). Both were expressed as mg of GAE/g and mg Eq. of quercetin/g.

### 2.4 Animals and treatments

One hundred and fifty rats at the age of 4 weeks (*n* = 150; weight = 80–90 g/rat) were purchased from the lab animal breeding center of the University of Veterinary and Animal Sciences, Lahore. Rats were kept in cages under control environmental temperature of 23 ± 2°C. Rats were free to access a standard diet and water throughout the experimental period. Rats were acclimatized for 1 week before the start of the experiment. Dexamethasone was injected subcutaneously at the dose of 0.5 mg/kg body weight on daily basis to induce oxidative stress (Xia et al., 2010).

Experimental rats were divided into two groups of 70 and 80 rats, which were treated for 15 days. Group 1: no treatment or untreated rats, while Group 2: 40 mg/kg lead acetate intraperitoneal every other day (Zhang et al., 2013).

After 20 days, nonlead exposed rats or lead untreated rats were divided into seven groups of 10 rats per group, which was treated for 10 days, as shown in Table 1. The lead‐treated rats were divided into eight groups of 10 rats per group and given treatment for 10 days, as shown in Table 1. At the end of the experiment, all animals were sacrificed and blood samples were collected from the heart. The liver and kidney were collected for further analysis.

**TABLE 1:** Study design.

Sr.	Groups (*n* = 10 rats/groups)	Description
1	Control	Normal Saline I.P.
2	Pb exposed	Lead Acetate 40 mg/kg I.P.
3	Pb + Sil	Lead Acetate 40 mg/kg I.P. & Silymarin 400 mg/kg P.O.
4	Aq‐200 mg/kg	200 mg/kg aqueous extract P.O.
5	Aq‐400 mg/kg	400 mg/kg aqueous extract P.O.
6	Aq‐600 mg/kg	600 15 mL/kg aqueous extract P.O.
7	Et‐200 mg/kg	200 mg/kg ethanolic extract P.O.
8	Et‐400 mg/kg	400 mg/kg ethanolic extract P.O.
9	Et‐600 mg/kg	600 15 mL/kg ethanolic extract P.O.
10	Pb + Aq‐200 mg/kg	Lead acetate 40 mg/kg I.P. & 200 mg/kg aqueous extract P.O.
11	Pb + Aq‐400 mg/kg	Lead acetate 40 mg/kg I.P. & 400 mg/kg aqueous extract P.O.
12	Pb + Aq‐600 mg/kg	Lead acetate 40 mg/kg I.P. & 600 mg/kg aqueous extract P.O.
13	Pb + Et‐200 mg/kg	Lead acetate 40 mg/kg I.P. & 200 mg/kg ethanolic extract P.O.
14	Pb + Et‐400 mg/kg	Lead acetate 40 mg/kg I.P. & 400 mg/kg ethanolic extract P.O.
15	Pb + Et‐600 mg/kg	Lead acetate 40 mg/kg I.P. & 600 mg/kg ethanolic extract P.O.

### 2.5 Measurement of Pb in liver and kidney homogenates

The Pb concentration was measured in liver and kidney homogenates by flame atomic absorption spectroscopy (FAAS). The liver and kidney samples were dried at 65°C for 24 h to prepare their homogenates. The samples were then heated at 500°C for 12–18 h in a muffle furnace (ISOtemp). The samples were digested by heating a slow boil in 1 mL concentrated hydrogen chloride (10M, Fisher Scientific) and 3 mL concentrated nitric acid (6M, Fisher Scientific) for 4–7 h. Samples were boiled until 0.5–1 mL of sample remained after adding one drop of hydrogen peroxide. Then, 15–25 mL water was added to samples, and an absorbance reading was taken through FAAS at 285 nm. Standard curves were prepared using commercially available standards (Sigma). BSA 1577A and Tort‐2 (NIST, Gaithersburg, MD, USA) were used as controls to digest the tissues.

### 2.6 In vitro and in vivo DPPH free radical scavenger assay

DPPH free radical scavenger assay was performed to determine the in vitro and in vivo antioxidant activities. The concentration of solutions was as follows: 1 g of extracts was dissolved in methanol to prepare 10 mg/mL stock solution. The serial dilution was prepared, and the concentrations were as follows: 10, 50, 100, 200, and 400 μg/mL. Then, 0.5 mL plant extract was mixed with 3.6 mL DPPH solution and one was taken as control. All prepared samples were vortexed for 1–2 min and incubated in dark for 30 min at room temperature. All the samples were performed in triplicate. The absorbance was determined at 517 nm and the DPPH free radical was calculated by using the formula as follows:

$$\%\;\text{Radical}\;\text{scavenging}\;\text{activity}=\left(\left[\mathrm{AC}-\mathrm{AS}\right]/\mathrm{AC}\right)\times 100.$$

“AC” absorbance of the control and “AS” absorbance of samples.

Two hundred microliter of liver and kidney homogenate was taken from treated group rats and mixed with an equal amount of acetonitrile to make the depolarized serum. Then incubated for 2 min at room temperature and centrifuged. The 50 μL deproteinized serum was added to a test tube with 1840 μL methanol and 10 μL DPPH solution. At 517 nm, the absorbance was determined by vortexing for 1 min and incubating in the dark for 30 min. The DPPH radical was calculated by using the formula as follows:

$$\%\;\text{Radical}\;\text{scavenging}\;\text{activity}=\left(\left[\mathrm{AC}-\mathrm{AS}\right]/\mathrm{AC}\right)\times 100.$$

### 2.7 Antioxidant activities through FRAP assay

The ferric‐reducing ability of plasma (FRAP) assay is one of the important assays for the determination of antioxidants based on the reduction of ferric‐tri‐pyridyl triazine (Fe3+‐TPTZ) complex to ferrous tri‐pyridyl triazine (Fe2+‐TPTZ). The blue color was shown at 593 nm and the change in absorbance indicated antioxidant capacity in the liver and kidney. Homogenization of the liver and kidney was conducted in 0.2 M sucrose and 5 mM DTT buffer at pH 7.4 containing 0.25 M Tris buffer. The results are expressed as Trolox equivalents.

### 2.8 Biochemical assay

The activities of blood ALAD, ALT, AST, and AlP level were determined by using available commercial kits, as previously used by Xia et al. (2010).

### 2.9 Lipid peroxidation

Lipid peroxidation will be determined by measuring the malondialdehyde (MDA). Standard was prepared from 1,1,3,3‐tetra ethoxy propane of various concentrations 0.00, 1.25, 2.5, 5.0, 10.0, 25.0, and 50.0 μmoL/L. Reaction mixture 2 comprises of the sample (100 μL), 8.1% sodium dodecyl sulfate (200 μL), 20% acetic acid (750 μL) of 3.5 pH, and 0.8% aqueous TBA solution (750 μL). 350 μL of distilled water is added to the mixture and heated for 1 h at 95°C. Cooling with water, 1.5 mL of n‐butanol and 500 μL of distilled water were added and shaken vigorously. After centrifuging for 10 min at 1968 *g*, the absorbance was measured at 532 nm.

### 2.10 Catalase activity

Catalase activity was evaluated by using a technique previously described by Cao et al. (2014). Solutions used for this purpose were substrate containing 65 μmol hydrogen peroxide in sodium‐potassium phosphate buffer (PBS), PBS, and 32.4 mmol ammonium molybdate to stop the reaction. Three blanks were prepared. Blank‐1 (B1) contained substrate (500 μL), molybdate (500 μL) and serum (50 μL); blank‐2 (B2) contained substrate (500 μL), molybdate (500 μL), and PBS (50 μL); and blank‐3 (B3) contained PBS (500 μL), molybdate (500 μL), and PBS (50 μL). The sample contained substrate (500 μL), molybdate (500 μL), and serum (50 μL). One‐minute incubation was given to the sample at 37°C and absorbance was measured at 405 nm in a spectrophotometer. The following formula was used for calculations.

$$\text{Serum}\;\text{catalase}\;\text{activity}\;\left(\mathrm{KU}/L\right)=\frac{A\;\left(\text{Sample}\right)-A\;\left(B1\right)}{A\;\left(B2\right)-A\;\left(B3\right)}\times 271$$

### 2.11 Histopathological analysis

The liver and kidney samples were fixed in 10% formalin for histopathological analysis. The histological examination was carried out by cutting sections into 5 mm thick and staining them with hematoxylin and eosin (H&E).

### 2.12 Anti‐inflammatory activity through carrageenan‐induced paw edema

Anti‐inflammatory activity of *Argyrolobium roseum* (Camb.) Jaub & Spach extracts was assessed by using carrageenan‐induced rat paw edema. The treatment of Pb and different doses of *Argyrolobium roseum* (Camb.) Jaub & Spach extracts were given 30 min before inducing inflammation to respective groups of rats. The injection of 0.1 mL carrageenan suspension (1%) in normal saline was given to the supplanter surface of the right hind paw of rats to induce inflammation. A vernier caliper was used for measuring the linear circumference of the injected paw at 0, 1, 2, 3, and 4 h after injection.

### 2.13 Antipyretic activity through yeast‐induced pyrexia assay

Antipyretic activity of *Argyrolobium roseum* (Camb.) Jaub & Spach extracts and Pb‐induced rats were assessed through the yeast‐induced pyrexia method. The basal temperature of the groin region of rats was measured with the help of a digital clinical thermometer. Thereafter, a subcutaneous injection of yeast (15% w/v, 10 mL/kg) was given to the rats to induce pyrexia. After 24 h, the peak pyrexia temperature was measured, and doses of untreated control, Pb treatment, and different dose rates of *Argyrolobium roseum* (Camb.) Jaub & Spach extracts were administered.

### 2.14 Statistical analysis

Data were presented as means ± SE. Data were analyzed by one‐way ANOVA and significant data were analyzed using Duncan's multiple‐range tests among groups by using SPSS (version 20). *p* < .05 was kept as a significant value.

## 3 RESULTS

### 3.1 Effects of *Argyrolobium roseum* (Camb.) Jaub & Spach extracts on Pb concentration in liver and kidney homogenate

**FIGURE 1:**
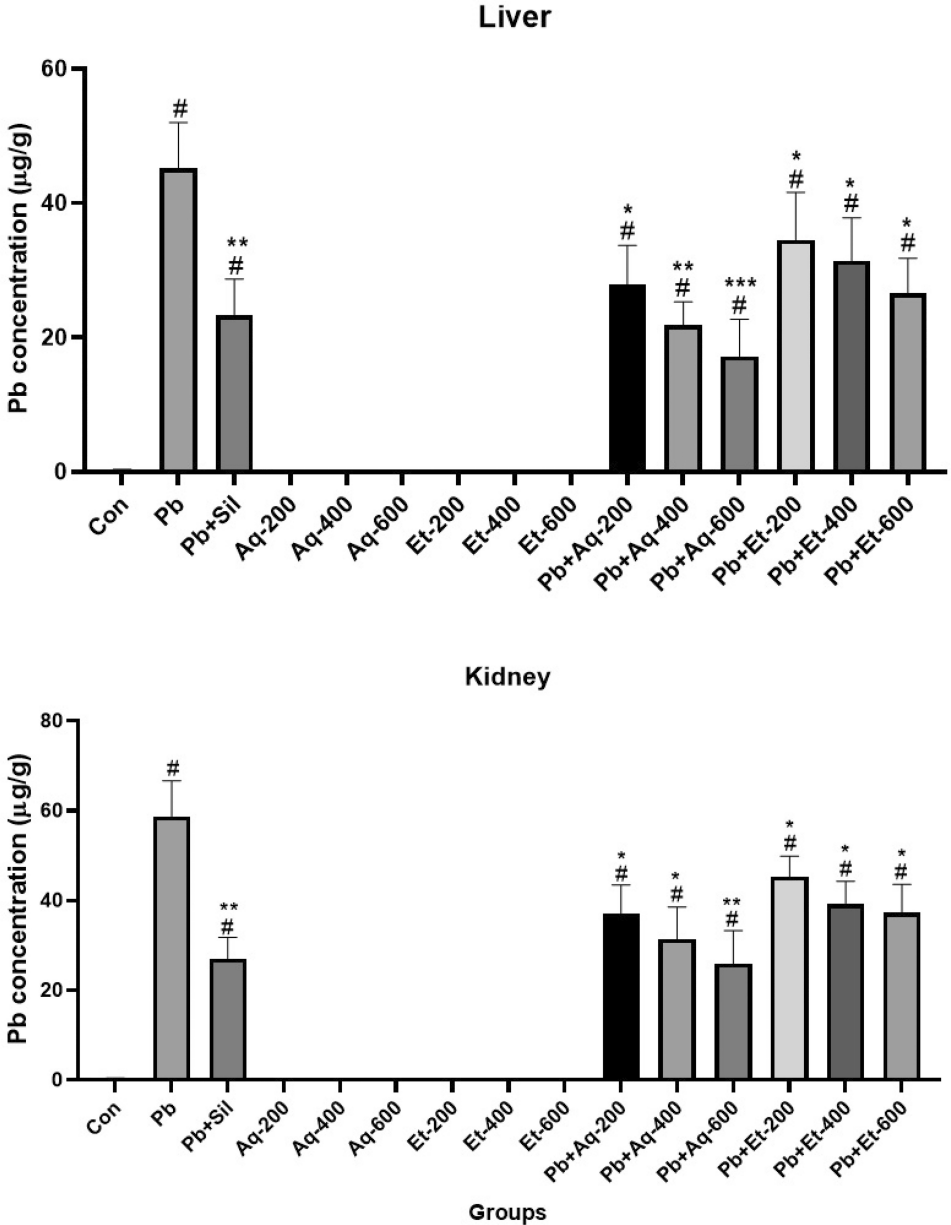
Effects of *Argyrolobium roseum* (Camb.) Jaub & Spach extract on concentrations of Pb in liver and kidney homogenate. Aq (Aqueous extract), Et (Ethanolic extract), and Sil (Silymarin), ^#^ *p* < .01 compared with Pb‐exposed group. ^##^ *p* < .001 compared with Pb‐exposed group. *p < .01 compared with control group (non‐lead exposed group). **p < .001 compared with control group (non‐lead exposed group). ***p < .0001 compared with control group (non‐lead exposed group).

The effect of *Argyrolobium roseum* (Camb.) Jaub & Spach extract treatments on the concentration of Pb in the liver and kidney homogenate is shown in Figure 1. The Pb concentration was significantly raised in liver and kidney homogenates (45.2 ± 6.8 and 58.8 ± 7.9, respectively; *p* < .0001) as injection of Pb acetate given i.p. in rats. The treatments of aqueous and ethanolic extracts of *Argyrolobium roseum* (Camb.) Jaub & Spach were significantly reducing the Pb concentration in the liver and kidney homogenates. The high‐dose treatment group (Pb + Aq‐600 mg/kg) efficiently reduced (*p* < .001) Pb concentration in both liver and kidney homogenates than Pb‐exposed rats and all other treatment rats.

### 3.2 Effect of *Argyrolobium roseum* (Camb.) Jaub & Spach extract on histological changes in liver and kidney

**FIGURE 2:**
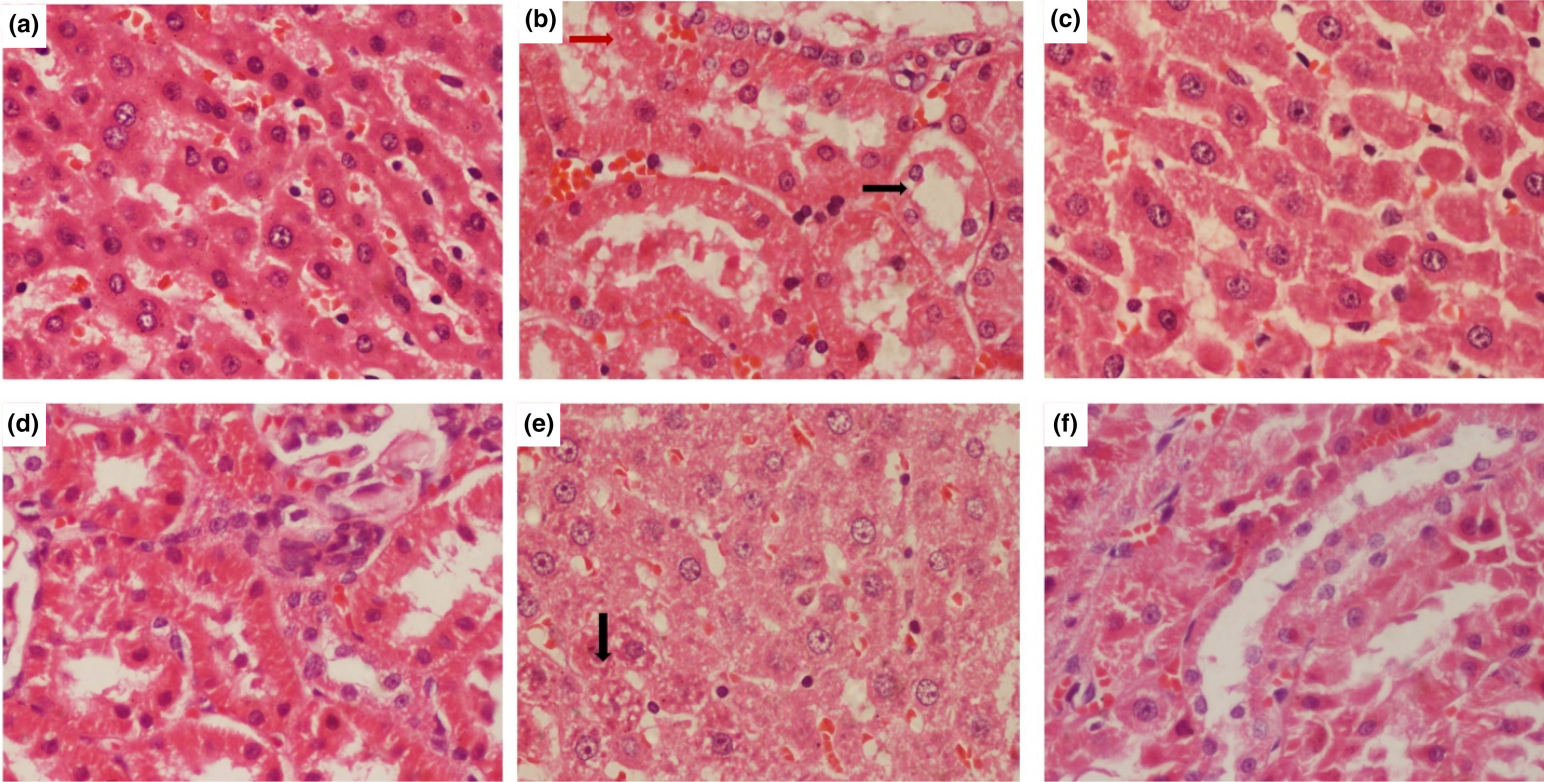
Effects of *Argyrolobium roseum* (Camb.) Jaub & Spach extract concentrations treatment on histopathological changes in rats' liver and kidneys stained with H&E.

The liver and kidney histological examination was used to evaluate the pharmacological effect of aqueous and ethanolic extract of *Argyrolobium roseum* (Camb.) Jaub & Spach on Pb‐induced injury, as shown in Figure 2. Histopathological results showed that Pb treatment caused noticeable morphological changes in rat liver, including cellular swelling and coagulative necrosis in the hepatocytes and leukocytes (Figure 2b). However, these morphometric changes were not present in the liver of control or Pb untreated rats (Figure 2a). The 600 mg/kg aqueous extract of *Argyrolobium roseum* (Camb.) Jaub & Spach + Pb–treated rats exhibited a protective effect on hepatocytes cells against Pb‐induced liver injury (Figure 2c).

There were visible histological changes in the kidney cortex of rats treated with Pb, acute peritubular congestion, mild cellular swelling, and coagulative necrosis in proximal tubular epithelium cells, as well as decreasing glomeruli (Figure 2e). Although the 600 mg/kg aqueous extract of *Argyrolobium roseum* (Camb.), the proximal tubular epithelium of rats treated with lead was slightly enlarged, whereas the glomeruli were almost normal (Figure 2f).

### 3.3 Antioxidant activity of liver homogenate

**FIGURE 3:**
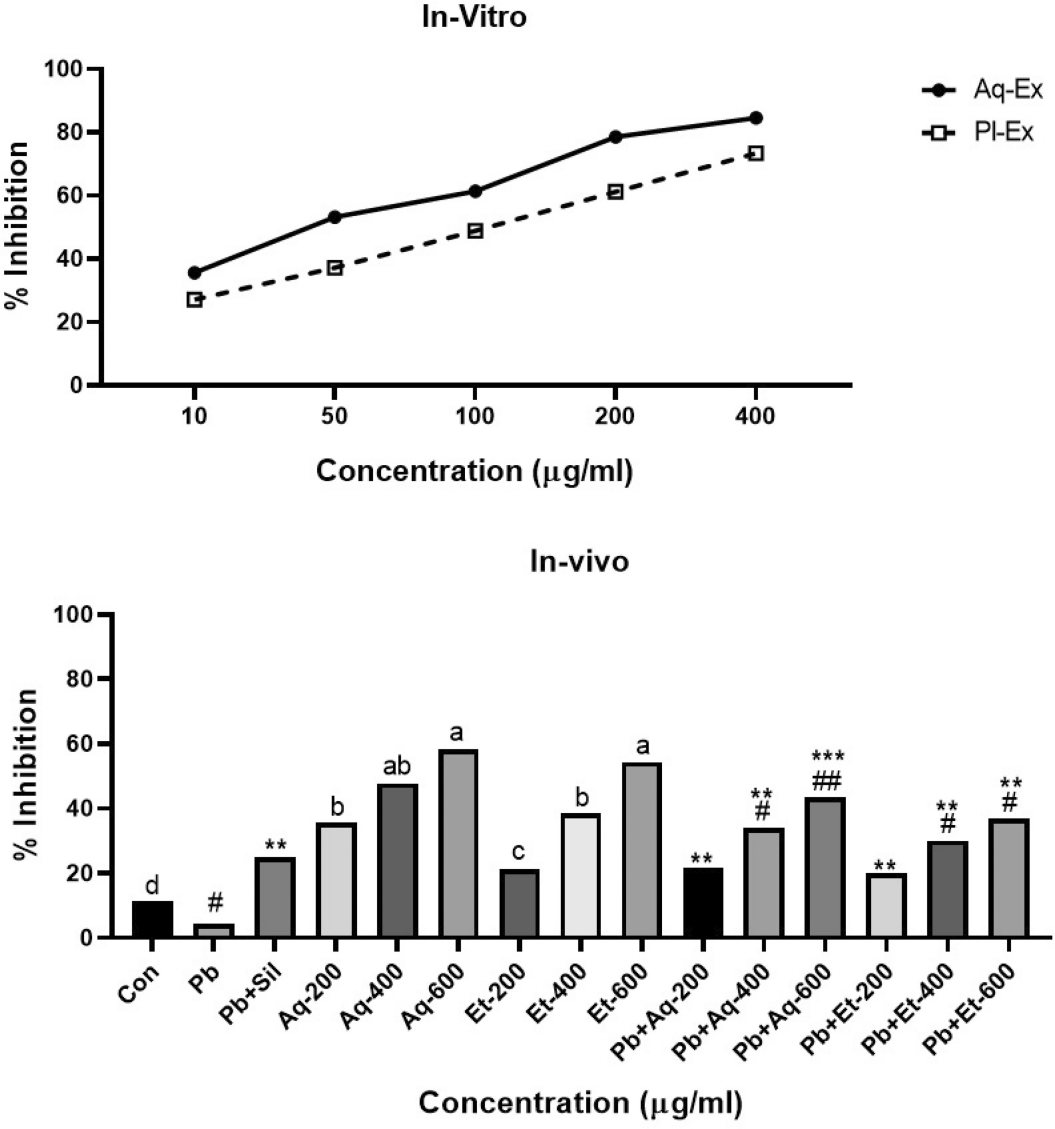
Shows in vitro and in vivo radical scavenging activity of *Argyrolobium roseum* (Camb.) Jaub & Spach extract concentrations through DPPH assay. Aq (Aqueous extract), Et (Ethanolic extract), and Sil (Silymarin), ^#^ *p* < .01 compared with Pb‐exposed group. ^##^ *p* < .001 compared with Pb‐exposed group. *p < .01 compared with control group (non‐lead exposed group). **p < .001 compared with control group (non‐lead exposed group). ***p < .0001 compared with control group (non‐lead exposed group).

The in vitro and in vivo antioxidant activity against free radicals was determined through DPPH free radical scavenging assay, while the total reducing ability in liver homogenate was assayed through the FRAP method. The inhibition results in in vitro antioxidant activity against DPPH free radical scavenger at various concentrations of aqueous and plant extract of *Argyrolobium roseum* (Camb.) Jaub & Spach are shown in Figure 3a. The in vitro percent inhibition was significantly increased by increasing the concentrations of both aqueous and plant extracts (35.63%, 53.21%, 61.38%, 78.51%, and 84.51% in aqueous extracts, while 27.12%, 37.23%, 48.91%, 61.21%, and 73.34% in plant extracts). This increased percent inhibition showed the increased antioxidant potential of *Argyrolobium roseum* (Camb.) Jaub & Spach.

The in vivo percent inhibition of free radicals by *Argyrolobium roseum* (Camb.) Jaub & Spach extracts in liver and kidney homogenate is shown in Figure 3b. Significant (*p* < .001) differences were observed in DPPH free radical scavenging in liver and kidney homogenates of rats which were treated with 600 mg/kg alone aqueous or ethanolic extracts. The highest DPPH free radical decomposition of liver and kidney homogenate was observed in the Pb + Aq‐600 mg/kg group as compared to other treated groups.

**FIGURE 4:**
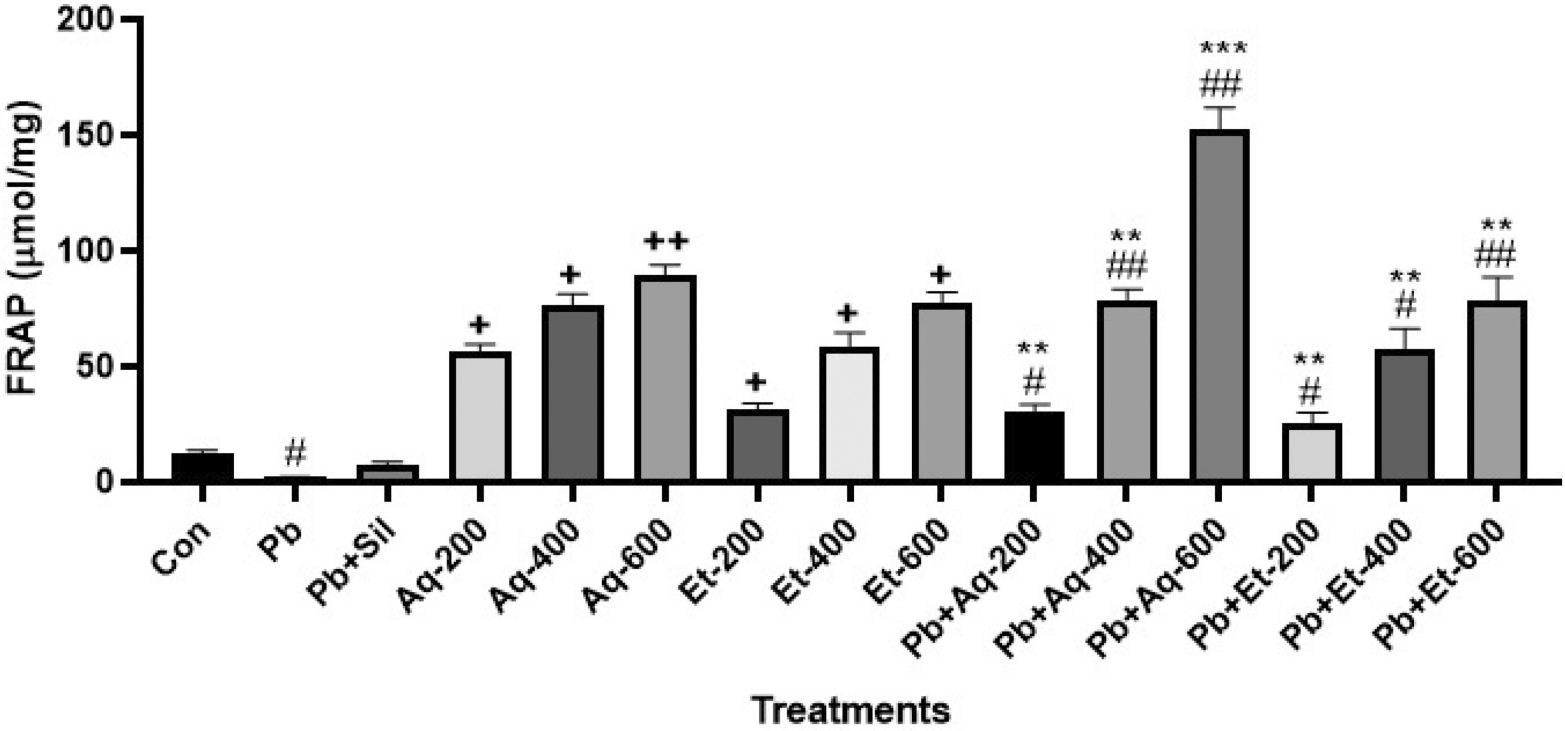
Shows radical scavenging activity of *Argyrolobium roseum* (Camb.) Jaub & Spach extract concentrations through FRAP assay. Aq (Aqueous extract), Et (Ethanolic extract), and Sil (Silymarin), ^#^ *p* < .01 compared with Pb‐exposed group. ^##^ *p* < .001 compared with Pb‐exposed group. *p < .01 compared with control group (non‐lead exposed group). **p < .001 compared with control group (non‐lead exposed group). ***p < .0001 compared with control group (non‐lead exposed group), ^+^ *p* < .01 and ^++^ *p* < .001 compared with control group.

The results of antioxidant activity in the liver were assayed through the FRAP method, which is shown in Figure 4. The results showed that antioxidant activity based on FRAP value was significantly high in the liver of rats when treated with Pb + Aq‐600 mg/kg *Argyrolobium roseum* (Camb.) Jaub & Spach than any other treatment groups (152.1 ± 7.89; *p* < .001).

### 3.4 Effect of oxidative stress on liver homogenates and antioxidant parameters

The level of antioxidant parameters in liver homogenates is shown in Table 2. The MDA level was increased in liver homogenates of rats after Pb exposure as compared to the control (21.3 ± 2.13 nmol/mg; *p* < .001). The level of MDA was significantly decreased (*p* < .01–.001) after treatment of aqueous and ethanolic extract of *Argyrolobium roseum* (Camb.) Jaub & Spach than Pb exposure group. The highest decreased MDA level was found in liver homogenate of Pb + Aq‐600 mg/kg rats (11.2 ± 1.51 nmol/mg; *p* < .001). The GSH and CAT level was significantly reduced in liver homogenate after the Pb exposure. Its level tended to be normal (*p* < .001) after treatment of Aq‐400, 600, and Et‐600 mg/kg *Argyrolobium roseum* (Camb.) Jaub & Spach. The MDA, GSH, and CAT levels were unchanged after treatment of Aq‐200–600 and Et‐200–600 mg/kg *Argyrolobium roseum* (Camb.) Jaub & Spach individually.

**TABLE 2:** Effects of *Argyrolobium roseum* (Camb.) Jaub & Spach extract on MDA, GSH, GSSG, and Cat activity, indicative of liver oxidative stress in lead‐exposed rats.

Treatments	MDA (nmol/mg)	GSH (nmol/mg protein)	CAT (μmol/mg protein)
Control	7.1 ± 0.76	23.9 ± 4.32	62.1 ± 4.32
Pb exposed	21.3 ± 2.13**	11.3 ± 2.18**	28.9 ± 3.91***
Pb + Sil	17.8 ± 3.31**	14.5 ± 1.91^#,^**	42.2 ± 2.15^#,^**
Aq‐200 mg/kg	5.3 ± 0.91	22.1 ± 3.32	61.2 ± 1.98
Aq‐400 mg/kg	4.5 ± 0.63	23.6 ± 4.47	62.4 ± 3.71
Aq‐600 mg/kg	3.2 ± 0.43	25.4 ± 2.89	63.1 ± 2.51
Et‐200 mg/kg	6.7 ± 0.71	21.3 ± 1.94	60.5 ± 2.33
Et‐400 mg/kg	5.2 ± 0.38	23.6 ± 3.73	61.4 ± 2.85
Et‐600 mg/kg	4.7 ± 0.61	24.7 ± 2.67	63.1 ± 2.19
Pb + Aq‐200 mg/kg	16.6 ± 2.11^#,^**	15.7 ± 3.65^#,^**	41.4 ± 3.67^#,^**
Pb + Aq‐400 mg/kg	14.2 ± 3.41^#,^*	17.5 ± 2.19^##,^*	53.9 ± 2.39^##,^*
Pb + Aq‐600 mg/kg	11.2 ± 1.51^##,^*	18.9 ± 3.82^##,^*	58.4 ± 2.82^##^
Pb + Et‐200 mg/kg	18.5 ± 3.93**	15.1 ± 1.85^#,^**	34.7 ± 4.51***
Pb + Et‐400 mg/kg	17.3 ± 3.22**	16.7 ± 3.56^#,^**	43.8 ± 4.83^#,^**
Pb + Et‐600 mg/kg	14.6 ± 2.85^#,^*	18.2 ± 2.49^##,^*	50.7 ± 3.59^##,^*

*Note*: ^#^ *p* < .01 compared with Pb‐exposed group. ^##^ *p* < .001 compared with Pb‐exposed group. *p < .01 compared with control group (non‐lead exposed group). **p < .001 compared with control group (non‐lead exposed group). ***p < .0001 compared with control group (non‐lead exposed group).

Abbreviations: Aq, Aqueous extract; Et, Ethanolic extract; Sil, Silymarin.

### 3.5 Effect of oxidative stress on kidney homogenates and antioxidant parameters

The level of antioxidant parameters in kidney homogenates is shown in Table 3. The MDA level was increased in kidney homogenates of rats after Pb exposure as compared to the control (17.8 ± 1.03 nmol/mg; *p* < .0001). The level of MDA was significantly reduced after the treatment of Pb‐Aq‐600 mg/kg *Argyrolobium roseum* (Camb.) Jaub & Spach than Pb exposure, and all other treatment groups (8.8 ± 2.71; *p* < .001), but slightly higher (*p* < .01) than control. The GSH and CAT level was significantly reduced in kidney homogenate after the Pb exposure. The level of GSH tended to be normal (*p* < .01) after treatment of aqueous and ethanolic extract of *Argyrolobium roseum* (Camb.) Jaub & Spach than Pb‐exposed group and slightly higher (*p* < .01) than the control. However, the CAT was reversed to normal after treatment of aqueous and ethanolic extract of *Argyrolobium roseum* (Camb.) Jaub & Spach.

**TABLE 3:** Effects of *Argyrolobium roseum* (Camb.) Jaub & Spach extract on MDA, GSH, and Cat activity indicative of kidney oxidative stress in lead‐exposed rats.

Treatments	MDA (nmol/mg)	GSH (nmol/mg protein)	CAT (μmol/mg protein)
Control	4.3 ± 0.11	22.5 ± 2.82	46.1 ± 5.12
Pb exposed	17.8 ± 1.03***	13.3 ± 1.13**	31.5 ± 4.21**
Pb + Sil	14.3 ± 1.61^#,^**	16.5 ± 2.41^#,^*	41.1 ± 4.35^#^
Aq‐200 mg/kg	4.2 ± 0.53	22.1 ± 1.32	46.2 ± 1.58
Aq‐400 mg/kg	4.1 ± 0.43	22.9 ± 2.17	46.6 ± 3.41
Aq‐600 mg/kg	3.5 ± 0.73	23.4 ± 1.75	46.9 ± 2.51
Et‐200 mg/kg	4.7 ± 0.21	22.3 ± 2.24	45.5 ± 3.13
Et‐400 mg/kg	4.2 ± 0.33	22.6 ± 1.33	46.1 ± 4.55
Et‐600 mg/kg	3.8 ± 0.41	23.2 ± 2.31	46.3 ± 3.31
Pb + Aq‐200 mg/kg	14.6 ± 1.51^#,^**	16.7 ± 2.85^#,^*	40.4 ± 3.27^#^
Pb + Aq‐400 mg/kg	11.2 ± 2.11^#,^**	17.1 ± 1.69^#,^*	43.9 ± 4.19^##^
Pb + Aq‐600 mg/kg	8.8 ± 2.71^##,^*	19.4 ± 2.12^##,^*	44.4 ± 3.22^##^
Pb + Et‐200 mg/kg	15.3 ± 2.33^#,^**	16.1 ± 2.31^#,^*	39.7 ± 3.72^#^
Pb + Et‐400 mg/kg	13.4 ± 1.22^#,^**	16.8 ± 2.64^#,^*	40.8 ± 2.53^#^
Pb + Et‐600 mg/kg	11.6 ± 3.15^#,^**	17.6 ± 1.24^#,^*	42.7 ± 4.21^#^

*Note*: ^#^ *p* < .01 compared with Pb‐exposed group. ^##^ *p* < .001 compared with Pb‐exposed group. *p < .01 compared with control group (non‐lead exposed group). **p < .001 compared with control group (non‐lead exposed group). ***p < .0001 compared with control group (non‐lead exposed group).

Abbreviations: Aq, Aqueous extract; Et, Ethanolic extract; Sil, Silymarin.

### 3.6 Effect of Pb on liver function enzyme level

The serum ALAD, ALT, AST, and ALP level were reduced (*p* < .05) after Pb exposure in rats than the control group, as shown in Table 4. Meanwhile, the ALAD, ALT, AST, and ALP level were enhanced and tended to be normal after the Aq‐400 and Aq‐600 mg/kg *Argyrolobium roseum* (Camb.) Jaub & Spach in Pb‐exposed rats in comparison to other treatments group. However, the treatment of aqueous and ethanolic extract of *Argyrolobium roseum* (Camb.) Jaub & Spach individually does not affect ALT, AST, ALP, and ALAD levels.

**TABLE 4:** Effects of *Argyrolobium roseum* (Camb.) Jaub & Spach extract on Pb‐sensitive biochemical variables in Pb‐induced rats.

Treatments	ALAD nmol/min/mL	ALT units/L	AST units/L	ALP units/L
Control	7.43 ± 0.46	30.93 ± 2.32	112.1 ± 7.92	95.1 ± 4.32
Pb exposed	1.32 ± 0.13***	18.53 ± 2.18**	93.9 ± 5.11**	88.9 ± 3.91*
Pb + Sil	3.28 ± 0.31^#,^**	24.51 ± 1.91^#,^*	102.2 ± 8.15^#,^*	91.2 ± 2.15*
Aq‐200 mg/kg	7.34 ± 0.41	31.11 ± 3.32	111.2 ± 6.18	96.2 ± 1.98
Aq‐400 mg/kg	6.95 ± 0.63	30.26 ± 4.47	112.4 ± 6.73	96.4 ± 3.71
Aq‐600 mg/kg	7.27 ± 0.43	32.74 ± 2.89	113.1 ± 6.21	96.1 ± 4.71
Et‐200 mg/kg	7.57 ± 0.71	31.32 ± 1.94	110.5 ± 3.93	95.5 ± 2.93
Et‐400 mg/kg	6.98 ± 0.38	32.63 ± 3.73	111.4 ± 7.34	96.4 ± 3.25
Et‐600 mg/kg	7.37 ± 0.61	31.37 ± 2.67	113.1 ± 5.31	96.1 ± 5.19
Pb + Aq‐200 mg/kg	2.63 ± 0.21^#,^***	25.27 ± 3.65^#,^*	94.4 ± 3.67**	91.4 ± 5.87*
Pb + Aq‐400 mg/kg	3.12 ± 0.41^#,^**	27.57 ± 2.19^##,^*	103.9 ± 8.39^#,^*	93.9 ± 4.31^#^
Pb + Aq‐600 mg/kg	5.27 ± 0.51^##,^*	28.49 ± 3.82^##,^*	108.4 ± 6.44^##^	95.4 ± 7.12^#^
Pb + Et‐200 mg/kg	1.52 ± 0.53***	25.17 ± 1.85^#,^*	94.7 ± 4.51**	90.7 ± 4.51*
Pb + Et‐400 mg/kg	2.39 ± 0.22***	26.77 ± 3.56^##,^*	99.8 ± 4.33^#,^*	91.8 ± 3.59*
Pb + Et‐600 mg/kg	4.26 ± 0.53^#,^*	28.23 ± 2.49^##,^*	107.7 ± 3.59^##^	92.7 ± 5.22^#,^*

*Note*: ^#^ *p* < .01 compared with Pb‐exposed group. ^##^ *p* < .001 compared with Pb‐exposed group. *p < .01 compared with control group (non‐lead exposed group). **p < .001 compared with control group (non‐lead exposed group). ***p < .0001 compared with control group (non‐lead exposed group).

Abbreviations: Aq, Aqueous extract; Et, Ethanolic extract; Sil, Silymarin.

### 3.7 Anti‐inflammatory and antipyretic effect of *Argyrolobium roseum* (Camb.) Jaub & Spach

**FIGURE 5:**
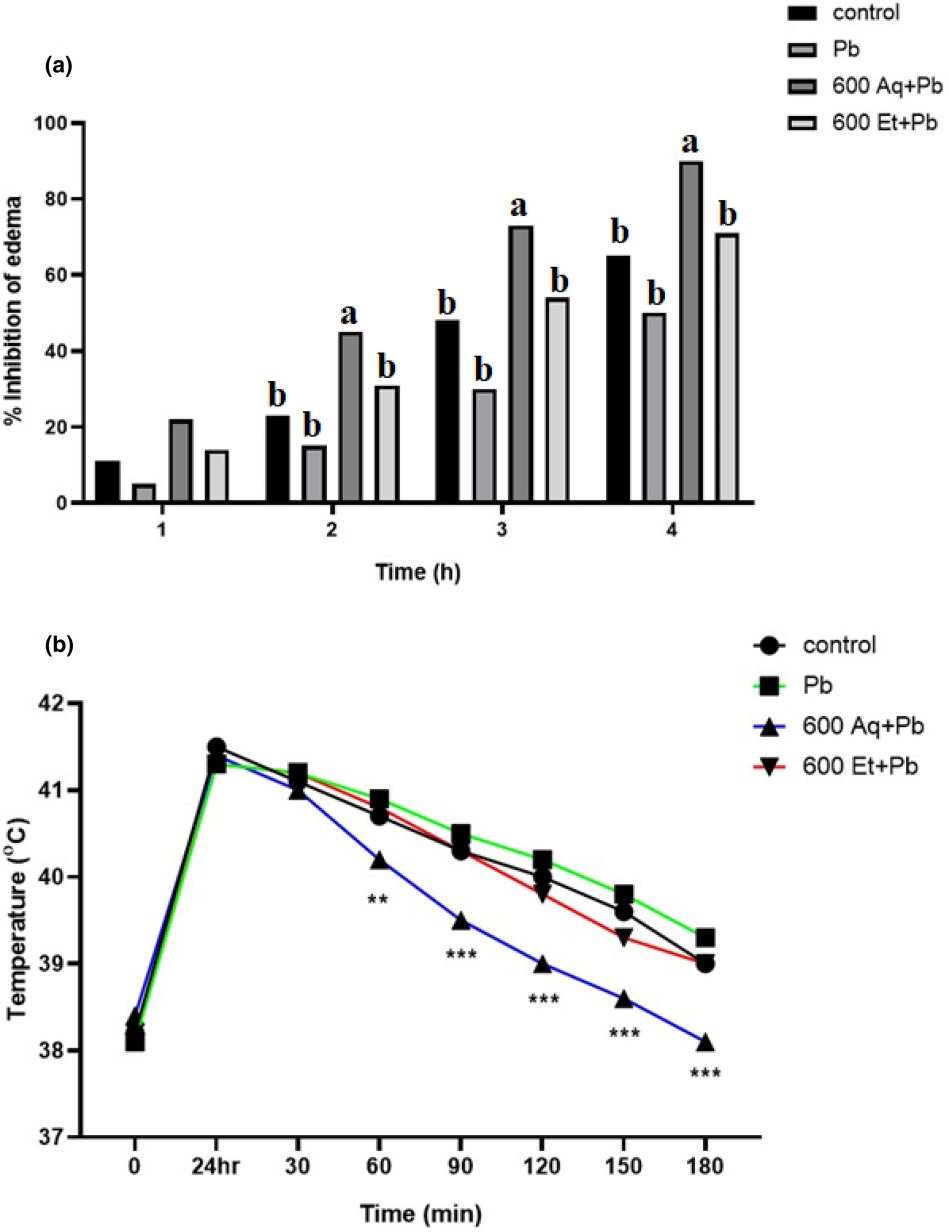
(a) Anti‐inflammatory activity of *Argyrolobium roseum* (Camb.) Jaub & Spach extract concentrations and (b) Anti‐pyretic activity of *Argyrolobium roseum* (Camb.) Jaub & Spach extract concentrations.

The anti‐inflammatory and antipyretic activity of *Argyrolobium roseum* (Camb.) Jaub & Spach was shown in Figure 5. The result showed that 600 mg/kg Aq + Pb exhibited significant (*p* < .001) anti‐inflammatory activity as compared to control and other treatment groups. At 3–4 h, 600 mg/kg Aq + Pb reduced edema by 73.5% and 90.1%, respectively. However, 600 mg/kg Et + Pb reduced edema by 75% at 4 h. The 600 mg/kg Aq + Pb significantly inhibited yeast‐induced pyrexia in rats. At 60 min post‐administration, a 32% (*p* < .01) decrease in yeast‐induced pyrexia improved to 50%–97% (*p* < .001) at 90–180 min post‐administration.

## 4 DISCUSSION

Lead is one of the most toxic and lethal metals for all mammals including livestock animals and humans (Barbosa et al., 2009). Pb is accumulated in the liver, kidney, bones, and other organs of the body within 1 h after intestinal absorption (Xia et al., 2010). The liver and kidney are both vital organs of the human body as well as of other animals. Therefore, many studies were conducted to remove Pb from body organs and also decrease the accumulation of Pb in the vital organ of the body. Occupational and environmental sources of exposure contribute to high Pb levels in many areas.

According to previous studies in mice and rats, Pb accumulation was observed in the liver and kidney of rats after treatment (Barbosa et al., 2009). The results of the current study showed that Pb concentration in both kidney and liver homogenates was significantly at a high level than in control groups. The high dose of aqueous extract of *Argyrolobium roseum* (Camb.) Jaub & Spach significantly decreases the Pb concentration in both kidney and liver homogenates. Similar results were found after the treatment of verapamil, nimodipine1, and Smilax glabra extract (Singh et al., 2018; Xia et al., 2010). The reason for the similarity in the result of the current study with previous reports may be due to increased phenolic content in the plant extract, which regulated or improves the excretion of Pb from the body (Lu et al., 2006; Zhang et al., 2009).

Liu et al. (2010) observed that Pb exposure damage the hepatocyte cell in the liver and increase apoptosis in rats and mice. According to our findings, induced Pb toxicity results in liver disruption and deleterious effects on liver cells. Previous reports showed that both kidney and liver are seriously affected by Pb exposure, including necrosis, cellular swelling, severe peritubular congestion, and mild cellular swelling and coagulative necrosis proximal tubular epithelial cells, as well as diminishing kidney glomeruli (Yin et al., 2008; Zhang et al., 2013). Similar findings were observed in the current study. In the current study, liver and kidney damage was reduced after the feeding of high‐dose aqueous and ethanolic extracts of *Argyrolobium roseum* (Camb.) Jaub & Spach. Similar findings were observed by Liu et al. (2010) that liver hepatic cells were in normal histology and function after the administration of Smilax glabra extract in high doses and good correlation with tissues antioxidant enzyme activities, plasma aminotransferase activities, and lipid peroxidation in mice. This protective effect of *Argyrolobium roseum* (Camb.) Jaub & Spach showed high anti‐apoptotic activity as previously observed in verapamil and nimodipine by Zhang et al. (2013).

The Pb toxicity leads to oxidative stress, and disturbance in pro‐oxidant and antioxidant levels in body organs (Wang et al., 2010). It also increased oxygen free radicals which disrobed mitochondrial structure (Xia et al., 2010). DPPH assay is one of the most efficient assays to determine the antioxidant activity of different chemicals. The researcher proposed that it is a relatively simple and economical approach (Singh et al., 2018). The current study showed that aqueous and ethanolic extracts of *Argyrolobium roseum* (Camb.) Jaub & Spach exhibited high percent scavenging free radical inhibitions at the lowest concentrations, and it was increased as the concentrations increase. Similar antioxidant activity was observed by using African *Sphenostylis stenocarpa* phenolic extracts, *Cassia fistula* seeds extract, and *Moringa pleifera* (Liqin et al., 2013). One possible explanation for their antioxidant activity is that redox properties may be present in these extracts, which act on reducing agents and singlet oxygen quenchers (Sridhar et al., 2014). The high antioxidant activity of aqueous and ethanolic extracts in the current study may be due to the high concentration of phenols, flavonoids, and tannin compounds which are strongly associated with redox reaction (Wang et al., 2010).

The FRAP assay was another assay that was used for the determination of antioxidant activity in the current study. Previously this assay was used for antioxidant activity in the liver of dogs, rats, mice, and other animals (Liqin et al., 2013). Mostly FRAP assay is used in heart disease of dogs (Cao et al., 2014). Therefore, the FRAP assay was used to assess the antioxidant activity of aqueous and ethanolic extracts of *Argyrolobium roseum* (Camb.) Jaub & Spach in rat's liver. Current results showed that antioxidant activity based on FRAP significantly increases in the liver of rats when rats were treated with a high dose of aqueous or ethanolic extracts of *Argyrolobium roseum* (Camb.) Jaub & Spach. That show, a high improvement in the antioxidant defense system of the liver in rats. This result confirms, that aqueous and ethanolic extracts of *Argyrolobium roseum* (Camb.) Jaub & Spach improve the liver oxidative status after Pb treatment. As a result, this study provides evidence for the hepatic antioxidant defense system being elevated by natural antioxidants when high‐dose aqueous extracts are used.

In Pb‐treated rats and mice, there is an elevation of biomarkers of oxidative stress (Zhang et al., 2009). When Pb is exposed to the liver, MDA levels and H_2_O_2_ levels increase, and then changes in fatty acid composition of the membrane are observed (Anantha et al., 2012). According to the present study, rats with lead exposure experienced oxidative stress, as measured by MDA levels in the liver compared to controls. The results of the current study showed that aqueous or ethanolic extracts of *Argyrolobium roseum* (Camb.) Jaub & Spach decreased MDA levels in the liver, therefore acting as a powerful antioxidant and free radical scavenger. A similar finding was reported when quercetin was used in mice (Boots et al., 2008). As a result of their higher diffusion in membranes, these extracts may have a high level of antioxidant activity, scavenging free radicals from multiple sites within the lipid bilayer, and decreasing the level of lipid peroxide in Pb‐treated rats (Muthukumaran et al., 2008).

Many antioxidant enzymes protect the liver from cellular damage. These include superoxide dismutase (SOD), catalase (CAT) and glutathione peroxidases (GPx) are critical antioxidant enzymes (Boots et al., 2008). The determination of these enzymes assesses the oxidative stress in the liver cells. In literature showed that Pb disturbed the antioxidant activities by the disturbance in the SH groups in these enzymes. In the present research, the CAT level was decreased as the rats were treated by Pb which leads to oxidative stress. Pb breaks the ROS production and antioxidant system which leads to disturbed enzymes. Current results showed that after treatment with a high dose of aqueous extract of *Argyrolobium roseum* (Camb.) Jaub & Spach improved the CAT level in the Pb‐treated rats, which indicates powerful antioxidant activity like other antioxidants (Zhang et al., 2013).

The carrageenan‐induced paw edema test, one of the most widely used tests to evaluate the anti‐inflammatory properties of natural products, was used to determine the anti‐inflammatory activity of extracts in the present study. Current result showed that 600 mg/kg Aq + Pb exhibited significant (*p* < .001) anti‐inflammatory activity as compared to control and other treatment groups. At 3–4 h, 600 mg/kg Aq + Pb reduced edema by 73.5% and 90.1%, respectively, and however, 600 mg/kg Et + Pb reduced edema by 75% at 4 h. Similar findings were observed by (Pinheiro et al., 2011) when using Artemisia scoparia hydromethanolic extract (ASHME). We purposed to conduct further research in this regard to assess how lead affects genetic changes in reproduction and conception in animals and humans.

## 5 CONCLUSION

In recent research, Pb inductions have been shown to damage kidney and liver tissues. As a result of their accumulation in these organs of rats, they cause severe oxidative damage and functional disruptions. The treatment of aqueous extract of *Argyrolobium roseum* (Camb.) Jaub & Spach reduces the renal and hepatic damage in Pb‐induced rats, and it also decreases oxidative stress via improving antioxidant components and worked as a good anti‐inflammatory in Pb‐induced rats.

## AUTHOR CONTRIBUTIONS

**Naeem Rasool:** Writing – original draft (equal). **Muhammad Ovais Omer:** Writing – review and editing (equal). **Aqeel Javeed:** Investigation (equal); visualization (equal). **Muhammad Nawaz:** Writing – review and editing (equal). **Muhammad Imran:** Formal analysis (equal); software (equal). **Muzzamal Hussain:** Data curation (equal); methodology (equal). **Zarina Mushtaq:** Conceptualization (equal); resources (equal). **Entesssar AL Jbawi:** Supervision (equal).

## CONFLICT OF INTEREST STATEMENT

There is no conflict of interest.

## Data Availability

The data that support the findings of this study are available on request from the corresponding author.
